# Poverty and child health in the UK: using evidence for action

**DOI:** 10.1136/archdischild-2014-306746

**Published:** 2016-02-08

**Authors:** Sophie Wickham, Elspeth Anwar, Ben Barr, Catherine Law, David Taylor-Robinson

**Affiliations:** 1 Department of Public Health and Policy, University of Liverpool, Liverpool, UK; 2 Institute of Child Health, University College London, London, UK

**Keywords:** Children's Rights, Child poverty, Health inequalities, Child health professionals

## Abstract

There are currently high levels of child poverty in the UK, and for the first time in almost two decades child poverty has started to rise in absolute terms. Child poverty is associated with a wide range of health-damaging impacts, negative educational outcomes and adverse long-term social and psychological outcomes. The poor health associated with child poverty limits children's potential and development, leading to poor health and life chances in adulthood. This article outlines some key definitions with regard to child poverty, reviews the links between child poverty and a range of health, developmental, behavioural and social outcomes for children, describes gaps in the evidence base and provides an overview of current policies relevant to child poverty in the UK. Finally, the article outlines how child health professionals can take action by (1) supporting policies to reduce child poverty, (2) providing services that reduce the health consequences of child poverty and (3) measuring and understanding the problem and assessing the impact of action.

## Introduction

The latest figures suggest that in 2013–2014 there were 3.7 million children living in poverty in the UK—3 in every 10 children.^1^ Furthermore, levels of child poverty are rising. For the first time in almost two decades, child poverty in the UK increased in absolute terms in 2011–2012.^2^

Higher levels of child poverty are associated with worse child health outcomes. Children growing up in poverty in the UK experience a wide range of adverse child health and developmental outcomes, and are more likely to develop chronic conditions in childhood compared with more affluent children.^3^ It has been estimated that eliminating child poverty in the UK would save the lives of 1400 children under 15 years of age annually.^4^ Furthermore, the consequences of child poverty cost the UK economy £29 billion a year in 2013, up from £25 billion in 2008.^5^

**Figure 1:**
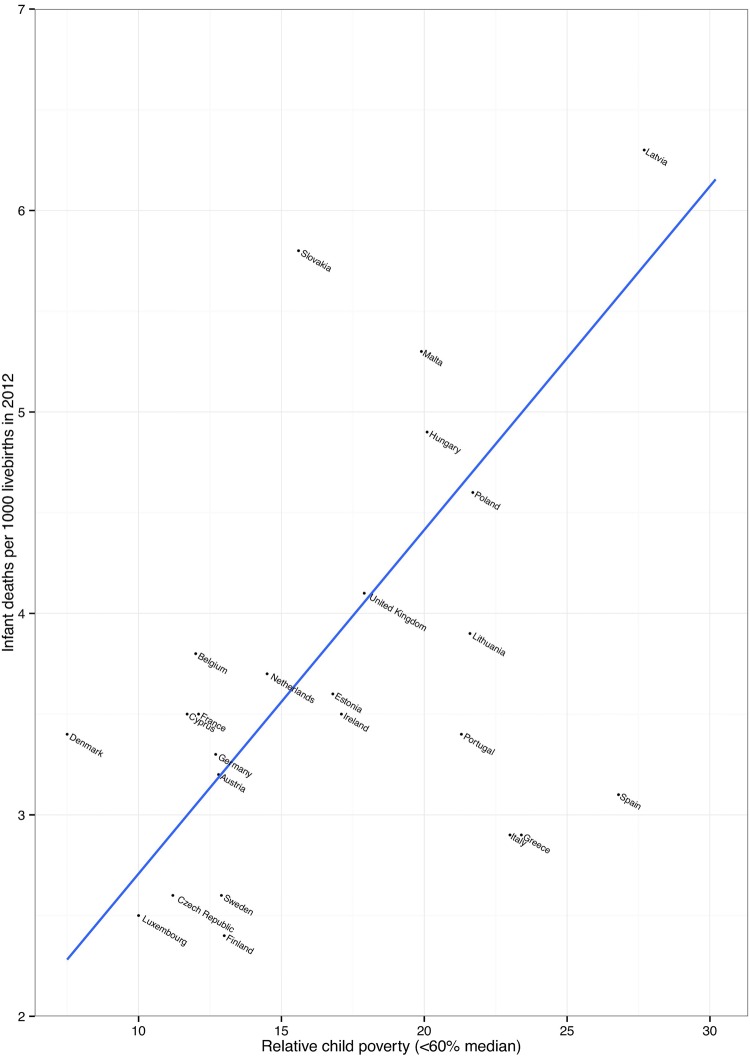
Child poverty and infant mortality in the Organisation for Economic Co-operation and Development (OECD) countries. Child poverty data are taken from EUROMOD figures, and infant mortality is taken from UNICEF (2014). EUROMOD, a European benefit-tax model and social integration.

The high level of poverty found in the UK is associated with many negative child health outcomes.^6^ For example, childhood mortality (aged 0–14) in the UK is significantly higher than similar countries in Europe.^7^ In children under five, the UK mortality rate is the highest in Western Europe, double that of Sweden.^8^ Figure 1 further shows that countries with a higher proportion of children living in relative poverty (below 60% median income) have higher infant mortality rates.

To assist child health professionals to engage in the debate about child poverty, here we outline some key definitions, review the links between child poverty and a range of health, developmental, behavioural and social outcomes for children,^9^ and provide an overview of current policies relevant to child poverty in the UK. Finally, we assess what further actions need to be taken and describe the important role that child health professionals can play.

## What is child poverty?

The theoretical underpinnings of ‘poverty’, how it is defined and measured are important as these concepts influence the strategies and policies chosen to address poverty. In 1979, Peter Townsend defined poverty as:

Individuals, families and groups in the population can be said to be in poverty when they lack resources to obtain the type of diet, participate in the activities and have the living conditions and amenities which are customary, or at least widely encouraged and approved, in the societies in which they belong. (ref. 10, p. 31)

This conception of poverty as being relative (rather than absolute) to a particular context recognises that standards of living change over time. The most widely used measure of relative poverty within the European Union is the proportion of individuals with household incomes less than a particular proportion of the current median of that population. For the purposes of international comparisons, UNICEF use a cut-off of 50%, whereas in the UK relative poverty is generally calculated as <60% of the median.^11^ ^12^ By contrast, absolute poverty is measured against a static threshold that only rises with inflation, even if society is becoming more or less prosperous. This measure indicates individuals living in poverty getting better or worse off in absolute terms.^12^ In practical terms, living on an income of <60% of the median means that many families struggle to meet basic needs like food, heating, transport, clothing and the extra costs of schooling like equipment and school trips.^13^

Being in receipt of income-related welfare benefits has also been used as a measure of poverty. In the UK, this can include being the recipient of income support, job seekers allowance, housing benefits, council tax benefits or working tax credit and child tax credit. Free school meal eligibility is a statutory benefit available to school-aged children from families who receive other qualifying benefits and is widely used as a measure of childhood disadvantage related to poverty, especially in educational analyses.^14^ This is often used as an area based measure, like the income deprivation affecting children index, which is the percentage of children aged 0–15 living in income-deprived households on the basis of receipt of various welfare benefits.^15^ Objective and subjective measures of material deprivation relating to lack of resources available to individuals that society deem important have also been used as child poverty measures. Subjective measures may include factors such as the extent to which children have birthday celebrations, appropriate clothes for all weather, holidays and parents with access to a car. In general, researchers have found similar patterns of association of poverty with child health outcomes whichever measure of poverty is used.^16^

Children can move in and out of poverty over the course of their lives. In the Millennium Cohort Study, a representative sample of children from the UK born in 2001, about half (47%) of children experienced relative poverty one or more times between the age of 9 months and 11 years, and 9% of children experienced persistent poverty (in all five waves of the study; S Wickham, E Anwar, B Barr, *et al*. Unpublished data: experiences of poverty in the UK Millennium Cohort Study).

## Health and social consequences of child poverty

Children living in poverty in the UK are more likely to:^9^

- die in the first year of life
- be born small
- be bottle fed
- breathe secondhand smoke
- become overweight
- suffer from asthma
- have tooth decay
- perform poorly at school
- die in an accident

**Figure 2:**
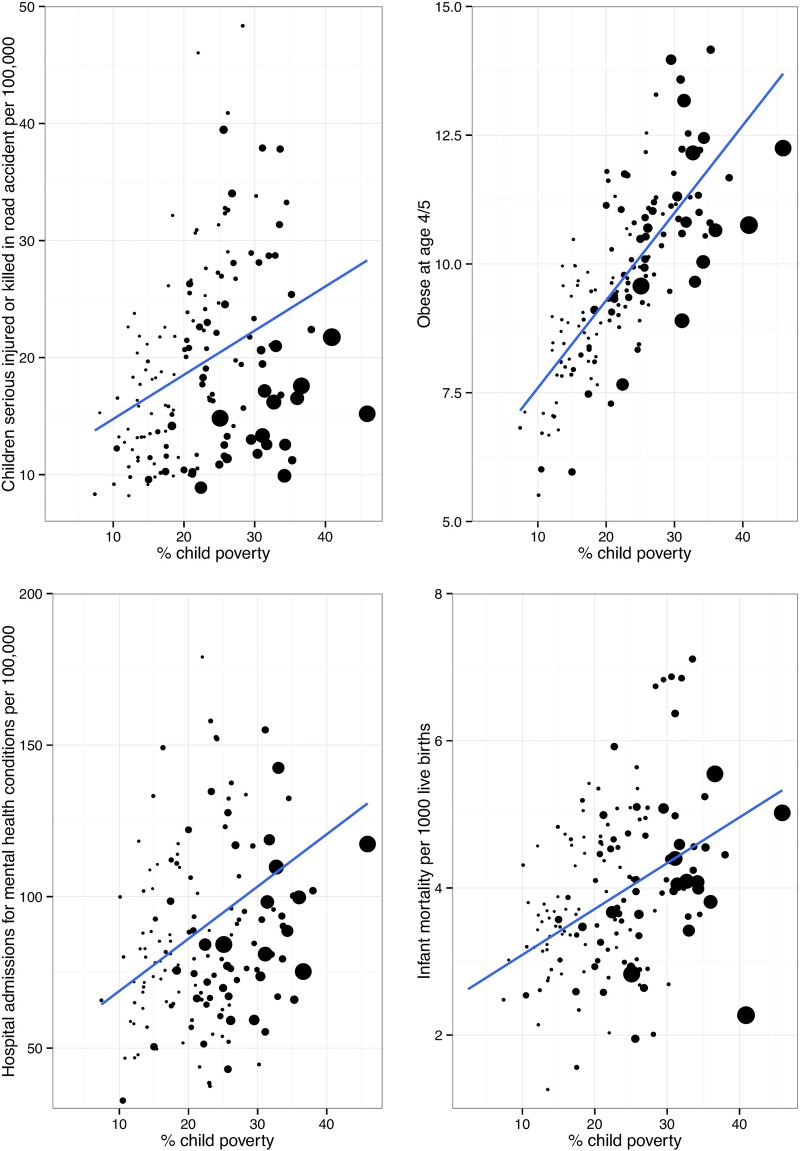
Child poverty and percentage of children seriously injured or killed in a road accident; obese at reception age; admitted to hospital with a mental health condition and infant mortality in Local Authorities in the UK. The size of the dot is proportional to population of each local authority. Data are from Public Health England (2015).

Even for children with genetic conditions like cystic fibrosis with no socio-economic bias in incidence, poorer children experience poorer outcomes, including worse growth, poorer lung function, higher risk of *Pseudomonas* infection, worse employment opportunities and ultimately poorer survival.^17^ ^18^ Figure 2 shows the association between levels of child poverty and a range of child health outcomes in local authorities in England.^19^

There has been some debate about the extent to which the relationship between poverty and health outcomes for children is causal or attributable to other factors. However, a recent systematic review of the literature concluded that a family's income makes a significant difference to children's outcomes: poorer children have worse cognitive, social-behavioural and health outcomes in part because they live in households with low incomes. This relationship was found to be independent of other factors that have been found to be correlated with child poverty (eg, household and parental characteristics).^20^ The review suggested that out of the 34 studies only 5 found no effect of child poverty on the various outcomes; this was mainly due to their methodological limitations.^20^ The authors highlight that longer durations of child poverty have a more severe effect on children's outcomes than short-term experiences of poverty.

Alongside these health-damaging impacts, living in poverty is associated with negative educational outcomes and adverse long-term social outcomes. Child poverty impacts on children's school readiness: by age five, children from the poorest fifth of homes in the UK are already on average over a year behind their expected years of development.^21^ By age 11, only three-quarters of the poorest children reach the government’s Key Stage 2 levels compared with 97% of children from the richest families.^22^ Only 21% of children from the poorest quintile, measured by parental socio-economic position, attain five good General Certificate of Secondary Education (grades A*– C) compared with 75% for their rich counterparts.^22^ Recent evidence suggests that child poverty is associated with structural differences in several areas of brain development, and this may account for the differences in academic achivements.^23^ Two recent studies from the USA show how child poverty influences the development of specific areas of the brain that are critical for the development of language, executive functions and memory.^23^ ^24^ This then impacts education prospects, job opportunities and future lifestyle choices.^25^

We know from longitudinal studies that children growing up in disadvantaged circumstances have a higher risk of death in adulthood across almost all conditions that have been studied, including mortality from stomach cancer, lung cancer, haemorrhagic stroke, coronary heart disease and respiratory-related deaths, accidents and alcohol-related causes of death.^26^ ^27^ These studies demonstrate that exposure to child poverty is a critical issue not just for child health, but also for adult health. Though the focus of this paper is on poverty, there is a social gradient in many of the health outcomes listed above, with greater social disadvantage leading to greater health impacts. This is powerful evidence that social and economic conditions do not just affect poor children but exert their influence across the entire social spectrum.^9^ ^28^ This has profound policy implications as the effect of policies on child poverty are then multiplied across children’s life courses. As children's lives unfold, the poor health associated with poverty limits their potential and development across a whole range of areas, leading to poor health and life chances in adulthood, which then has knock-on effects on future generations.^29^

## Research gaps

That poverty is bad for child health is not in doubt. What is unclear is how and when social disadvantage leads to ill health, that is, how it ‘gets under the skin’. Poverty has been highlighted as the most important social determinant of child health in high-income countries.^6^ ^30^ But poverty is likely to be the cause of wide-ranging effects on health exerted through a myriad of biological, behavioural, environmental and psychosocial mechanisms that are still not well understood.^8^ Poor health outcomes might be the result of cumulative exposure to disadvantage,^31^ or exposure during sensitive or critical periods, or both of these.^28^ For example, Seguin and colleagues have identified the importance of chronic cumulative poverty for outcomes such as asthma^32^ and obesity.^33^ Furthermore, poor health, particularly during critical periods of childhood and adolescence, may limit future development with subsequent effects on social position and health later in life.^25^ A better understanding is needed of the specific pathways through which exposure to adverse childhood socio-economic circumstances, and particularly poverty, affect specific health and social outcomes in particular conditions and contexts.^6^ ^20^ ^34^ Elucidating the mediating components of pathways will help identify times and circumstances that are amenable to intervention.

Cross-national comparisons may yield useful information in order to explain both the differences in child poverty rates in rich countries seen in figure 1 and how any policy differences impact on child health and well-being.^11^ Strategies to reduce child poverty and the consequences of child poverty generally involve three key components—early childhood education and care, income redistribution through the benefit and tax systems, and policies to increase the employment chances and wages of families living in poverty.^35^ While there is evidence that all three components are likely to be effective at reducing child poverty, less is known about whether some approaches are more likely to lead to greater health benefits than others. Further investigation is needed into the interaction between different policy approaches and the determinants of child health in order to prioritise policies that are likely to have the greatest impact not only on child poverty but also on child health.

### What is the UK currently doing about child poverty?

**Figure 3:**
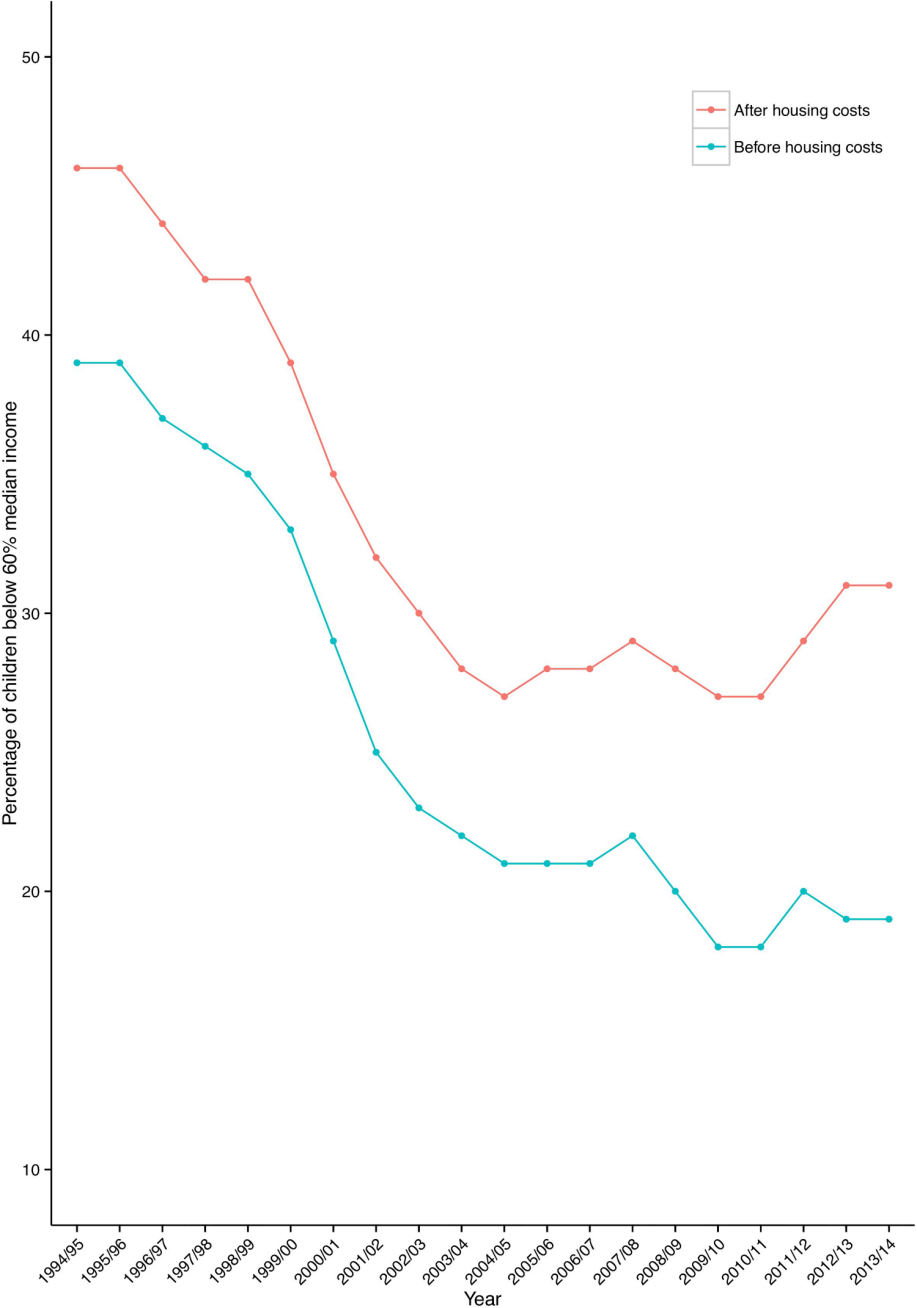
Trends in relative child poverty over time using data from Housing Below Average Income statistics.

Within the UK, several targets have been previously set to eradicate child poverty (see box 1 for details). Figure 3 shows the trends in child poverty over recent years. The UK was the first European country to systematically implement and evaluate policies aimed specifically at reducing child poverty.^36^ In particular, the Labour government set targets to reduce and eventually ‘eradicate’ child poverty, within 10–20 years. Though significant progress was made, the 2010 targets to halve child poverty were missed.

Box 1 **UK policy on child poverty**

*1999: Ending Child Poverty by 2020*: In 1999, the then Prime Minister Tony Blair made a commitment to halve child poverty by 2010 and eliminate child poverty by 2020. After many years of being a neglected issue, child poverty was on the political agenda.

Key actions to reduce child poverty included getting parents into work and a more progressive tax and benefits system (especially to those targeted at children such as child benefit and child tax credit).

*2010: The Child Poverty Act* was passed with cross-party support. The Act enshrined the child poverty promise in law and required the government to produce a national Child Poverty Strategy. The coalition government, elected in May 2010, pledged to maintain the goal of ending child poverty in the UK by 2020.

Although relative poverty fell substantially in the decade after the 1999 Tony Blair pledge to end child poverty, from 3.4 million children then to 2.6 million children, the 2010 child poverty targets were missed. Critics argued that not enough parents moved into work, and work did not pay as well as it should. The proportion of poor children who came from working households increased.

*2011: A new approach to child poverty: tackling the causes of disadvantage and transforming families’ lives 2011–2014* was published to fulfil the obligations under the Child Poverty Act 2010 to set out plans for tackling child poverty. It provided a framework for ending child poverty by 2020.

*2014: The child poverty strategy, 2014 to 2017* was published with two main aims to engineer a shift away from supporting families through income transfers towards tackling the root causes of poverty by enabling more parents to enter work and earn more. Second, to break the intergenerational cycle of poverty through raising the attainment of poor children so that they will be better off as adults.

The strategy was criticised by the Social Mobility and Child Poverty Commission for falling far short of what is needed and a missed opportunity to get back on track towards meeting its legal obligation to end child poverty by 2020. After a decade of falling levels, independent projections from both the Institute for Fiscal Studies (IFS) and the New Policy Institute (NPI) suggested that child poverty will increase by 2020.

*2015: The Welfare Reform and Work Bill* removes the government's duty to end child poverty by 2020 and changes the target for child poverty in the UK, moving away from a measure based on income to focusing on the ‘root causes’ of poverty such as unemployment and family breakdown.

There is concern that many of the proposed changes in the Bill will either push more children into poverty or limit the government's ability to properly monitor levels of child poverty across the UK. In particular, the income cap and changes to tax credits have also been strongly criticised for negatively affecting families with young children.

New definition of child poverty has also been criticised for having a moral and judgemental dimension. As there has also been an increase in the proportion of children in poverty living in a working family, critics argue that reporting on a measure focused on children in workless households will not get to the heart of understanding child poverty in the UK.

The current UK government has now abolished the Child Poverty Act and with it the target to eliminate child poverty by 2020. Alongside removing these targets, there has been a shift in how the UK government plans to measure child poverty from a focus on income-based indicators to factors related to ‘family breakdown, debt and addiction’^37^ outcomes that conflate the consequences of child poverty, with the cause—a lack of material resources.^38^

Recent analyses of current policies implemented in the UK in response to the economic crisis show that children are among the groups being hit hardest.^39^ We know that family incomes have fallen considerably during the recent economic downturn and have continued to decline as other economic indicators improve.^40^ Children's services are being disproportionately hit by current austerity measures, with early years budgets facing significant cuts.^41^

In the Summer Budget 2015, the chancellor announced more cuts to the welfare system to take the UK from a ‘low wage, high tax, high welfare economy’ to a ‘higher wage, lower tax, lower welfare country’.^41^ A report from the Joseph Rowntree Foundation analysis shows that it is poor children who are going to be hit hardest by these changes,^42^ with lone parents and families with children who depend on welfare support seeing their incomes significantly reduced. Although the controversial proposal to cut child tax credits was recently scrapped in the Chancellor's Autumn budget, these cuts will still be introduced later with the replacement of tax credits with a new system—Universal Credit.^43^ The government has argued that these cuts to in-work welfare benefits will be offset by the introduction of a higher minimum wage—referred to as a National Living Wage (NLW). The latest analyses, however, suggest that lone parents will still lose out, and for couples with children, both will have to work full time on the NLW to get close to a decent standard of living.^43^

### What needs to be done?

#### What child health professionals can do (both as individuals and as providers of health services)

All children have a right to the best possible health, as enshrined in the UN Convention on the Rights of the Child. The UK government, therefore, has a legal and moral responsibility to ensure that all children develop to their full potential. Based on recommendations made by the WHO Commission on the Social Determinants of Health, there are a number of ways that, as individuals or collectively, child health professionals should take action on the social determinants of health and reduce child poverty.^44^ ^45^

#### Support policies to reduce child poverty

Child health professionals and their professional associations can advocate for policy action on the social determinants that support parents’ capacity and ability to care for children.^46^ We need child health professionals to advocate for more equitable welfare reforms, with the test that they must protect children as the most vulnerable members of our society.^2^ This will include labour market, tax and transfer polices that aim to lift all families with children out of poverty.

We propose advocacy for policies that:^28^

- provide sufficient income support for an adequate quality of life for all families with children;
- provide affordable housing;
- provide affordable, high-quality early years childcare;
- provide affordable public transport;
- provide better social security support for families caring for children with chronic illness;
- prioritise active labour market programmes to achieve timely interventions to reduce long-term unemployment;
- tackle in-work poverty, through the introduction of a true living wage;
- support parents into employment in order to maximise household incomes.

#### Provide services that reduce the health consequences of child poverty

In order to reduce the consequences of poverty, a commitment to universal services and a focus on proportionate universalism (services provided to everyone, but with a scale and intensity that is proportionate to the level of need) that supports all children, particularly in the early years, is a critical and cost-effective investment, and these services should be protected.^47^ The Healthy Child Programme, for example, is based on a model of ‘proportionate universalism’.^48^

Some of the key actions recommended in the Marmot review^28^ and Field^49^ include:

- protecting investment in early years services;
- shifting expenditure towards the early years wherever possible;
- providing high-quality and consistent support and services for parents during pregnancy;
- provision of high-quality universal services in childhood;
- routine support to families through parenting programmes, children's centres and key workers, delivered to meet social needs;
- providing support so that all children can access a healthy diet in the early years;
- providing high-quality home visiting services;
- focusing on narrowing the educational attainment gap at all stages.

It is vital to take a whole family approach to the care of children, with appropriate involvement of the full range of social services support available to families living in disadvantaged circumstances that may help to mitigate some of the effects of poverty. Child health professionals need to speak up for their patients within management settings. At a community level, they need to advocate for a greater connectivity between general practitioner practices, hospitals, schools, community centres, benefit services and sure start centres to support parents to access all the benefits and services they are entitled to and work to reduce any stigma associated with using these services.^42^

#### Measure and understand the problem and assess the impact of action

Child health professionals have a key role in conducting high-quality research investigating the links between child poverty and health and investigating the impact of changes to service provision on health inequalities. This is a critical moment for children and families in the UK, facing changes to preventative services in the community at the same time as levels of child poverty increase. Important changes include the transfer of public health commissioning duties to local authorities (eg, the Health Visitor Implementation Programme) and the impact of cut backs to the role of children's centres in delivering the early years agenda.^50^ There is a clear need for a better understanding of the impacts of changes to services on the most disadvantaged, improved data and monitoring at an individual and population level.^2^

## Conclusions

A wealth of evidence demonstrates the toxic impact of child poverty: in physical changes in brain structure and poor health and life chances. Child poverty is rising, and the UK government has abolished plans to attempt to eradicate it. Child health professionals need to act as advocates for more equitable welfare reform in order to protect the most vulnerable in society. Children are often not in a position to speak out for themselves and for this reason are offered special protection under the United Nations Convention on the Rights of the Child.^51^ The arguments here are not just about the evidence. Reducing poverty and its impacts on children is morally and legally the right thing to do.
